# A typology of beliefs and misperceptions about the influenza disease and vaccine among older adults in Singapore

**DOI:** 10.1371/journal.pone.0232472

**Published:** 2020-05-06

**Authors:** Christopher L. Cummings, Wei Yi Kong, Jeanette Orminski

**Affiliations:** 1 Decision Analytica, LLC, North Carolina State University, Cary, North Carolina, United States of America; 2 Gillings School of Global Public Health, University of North Carolina—Chapel Hill, Chapel Hill, North Carolina, United States of America; 3 Wee Kim Wee School of Communication and Information, Nanyang Technological University, Singapore, Singapore; Uniwersytet Zielonogorski, POLAND

## Abstract

Access to the influenza vaccine pose little barriers in developed countries such as Singapore and vaccination against influenza is highly recommended for at-risk populations including older adults. However, vaccination rates are much lower than recommended despite the significant morbidity and mortality associated with the disease among this vulnerable population. Given timely goals to increase vaccine acceptance and uptake, we explored Singaporean older adults’ misperceptions about influenza disease and vaccine. Qualitative semi-structured interviews were conducted among 76 Singaporean adults aged 65 and above with no focus on a specific area in Singapore. Data were analyzed with grounded theory methods to understand participants’ attitudes, perceptions, and knowledge. We developed *in vivo* codes that reflect the verbiage used by participants and exhaustively catalogued themes through a constant comparison coding method. Focusing specifically on older adults’ misperceptions, seven main themes about influenza disease or vaccine emerged from our data analysis: *familiarity with influenza*, *misperceptions about influenza*, *personal susceptibility to influenza*, *familiarity with the influenza vaccine*, *misperceptions about the influenza vaccine*, *misperceptions about influenza vaccine usage*, and *opinions about and barriers to influenza vaccine uptake*. Notably, there is a lack of adequate knowledge and motivation in vaccinating against influenza among older adults in Singapore. Health communication needs to be more tailored toward older adults’ message processing systems and engage health professionals’ involvement in addressing the influenza disease and vaccine misperceptions identified in this study.

## Introduction

Preventing the spread of infectious diseases like influenza is a high-priority public health challenge. Influenza contributes to approximately 290,000 to 650,000 global deaths annually [1]. Seasonal influenza in Singapore presents significant health burdens with 15 influenza-related deaths among every 100,000 people and influenza-associated deaths are 11 times higher among the older adult segment than the general population [2]. Furthermore, influenza continues to pose threats of a future global pandemic and thus remains as a public health concern [3]. Vaccination remains the most effective practice to prevent influenza infection [4].

In general, vaccines have contributed a three percent annual decline in global deaths from vaccine-preventable diseases between 1990 and 2013 [5] and the prevention of 10 million global deaths between 2010 and 2015 [6]. For influenza, the Centers for Disease Control and Prevention [7] reports an overall reduction of 40% to 60% in infection risks through vaccination. While vaccines provide first-line disease prevention and are one of the most cost-effective initiatives to improve public health [8], immunization rates remain substandard and the literature investigating vaccine perceptions signals a pressing need to improve health literacies and health communication to dispel vaccine myths and promote immunization uptake around the world [9].

In Singapore, the threat of infectious disease spread is a high-priority public health concern. The high population density of the country exacerbates human-transmission of diseases like influenza and has been considered a potential “hot-spot” for disease outbreaks. This was indeed the case in 2003 when SARS (Severe Acute Respiratory Syndrome) was brought to Singapore by residents returning from overseas resulting in 238 infection cases and 33 deaths [10]. Later in 2009, 11% of the Singapore population were estimated to be infected with the H1N1 influenza virus in the first 10 weeks after the epidemic began in the United States [11]. Recent proposed changes to the Infectious Diseases Act to turn away unvaccinated travelers to Singapore [12] signal awareness from authorities to protect residents from foreign disease vectors. However, adequate influenza prevention requires substantial herd immunity fostered through individuals’ uptake of the seasonal vaccine. Unfortunately, experts’ recognition of the value of vaccines as an effective preventive tool may not be shared equally by the public. This sentiment toward vaccines is reflected in low immunization rates as Gan [13] reports that “adult vaccination rates in Singapore generally hover at below 20 percent” and “below 10 percent for those aged 50 to 69” [14]. Given that disease incidence rates could be reduced with increased vaccine uptake [15], nonvaccination against infectious diseases could instead pose risks of viral spread, increasing the probability of disease outbreaks.

Outbreaks of influenza in tropical regions such as Singapore are less defined than other parts of the world [e.g., 16, 17]. Many countries focus vaccine efforts in preparation for a specific “flu season” but in Singapore viral circulation occurs throughout the year with most cases falling between April to July and November to January, respectively. More than 2,800 daily cases of acute respiratory infections were reported to medical centers island wide in January 2017 and 2018 [18]. In 2006, Chow and colleagues [19] noted that influenza-associated deaths in Singapore between 1996 and 2003 were the highest among older adults aged 65 and above and further estimated that the yearly vaccine uptake rate among the elderly population is not greater than eight percent. More recent figures from the 2013 National Health Surveillance Survey also revealed that influenza vaccination coverage among Singaporeans aged 50 and above ranged from 14% to 20% [20].

While vaccines are important in preventing viral spread, vaccines are generally less efficacious for older users [21, 22]. Because older adults are at higher risks of hospitalization, mortality, and influenza-related complications including pneumonia [23, 24, 25], yearly vaccinations against influenza are nevertheless recommended [26]. Adults aged 65 and above are recommended to get the influenza vaccine and eligible to use their medical savings to pay for annual influenza vaccination that costs between 30 and 40 Singapore Dollars per dose (approximately 22 to 29 US Dollars) at any accredited hospitals, polyclinics, and selected private general practitioner clinics [26].

This study builds on recent investigations by Sundaram and colleagues [27] that noted challenges for influenza vaccine uptake among healthcare workers in Singapore. Our study furthers this foundational work by exploring the influenza disease and vaccination misperceptions of the most vulnerable population to the disease (older adults) with the hope that such information can contribute to improved disease prevention and treatment.

Given the pressing need for greater vaccine uptake among older adults, a more robust understanding of influenza vaccine perceptions is vital to improve health responses to reduce the disease burden within this high-risk group. By uncovering misperceptions about the influenza vaccine held by the elderly population through this qualitative approach, findings can be used to inform healthcare professionals on how vaccine uptake may be encouraged.

## Methods

### Study setting and design

Qualitative semi-structured individual interviews were employed to better understand older adults’ attitudes, knowledge, and perceptions about influenza disease and vaccination. A mixed team of five male and female undergraduate student interviewers received training that outlined details of the study and provided interviewing techniques with role plays and mock interviews to increase familiarity with the questionnaire and anticipated problems prior to data collection. Participants were invited to the study through telephone calls and emails with senior care facility directors who organized visiting time at their living facilities and local organizations. Individuals where then invited to participate in the study through direct communication *in situ*. Personal one-on-one interviews were conducted and audio-recorded in private settings at participants’ homes or senior care facilities respectively to maintain confidentiality and to provide a safe space for the participants to share their perceptions without judgment and influence of others. Following the interview protocol approved by Nanyang Technological University’s Institutional Review Board (IRB), all participants were first informed of study objectives as well as the interview procedures and length, and subsequently requested to provide their written informed consent prior to their participation.

### Participant selection

Given the study objective to identify salient misperceptions about influenza and vaccination among older adults in Singapore, our inclusion criteria required that participants be Singapore citizens or permanent residents aged 65 and above and be able to converse primarily using the English language. However, Singapore is a polylingual society and while English is widely spoken it is often mixed with terms and phrase usage from other languages. Among older adults in Singapore, the most common second language used is Mandarin Chinese. Thus, all interviewers in our project had to be bilingual and conversant in English and Mandarin Chinese. While interviews were conducted primarily in English, our project team anticipated short usage of Mandarin Chinese by our participants and were trained to conduct member checks [28]. In such cases where a participant used terms or phrases in Mandarin, our interviewers conducted member checks in both English and Mandarin to best match nuances in language use. Participants were compensated with either 20 Singapore Dollars (approximately 15 US Dollars) cash or gift voucher at the end of the interviews. 76 participants who conformed to our inclusion criteria completed the interview, thus providing a robust sample and dataset from to allow for greater descriptive findings and saturation.

### Instrument and data collection

We populated questions for our semi-structured interview protocol from reviews of influenza vaccine perception studies [e.g., 29, 30]. Interview topics included health information seeking behavior, influenza knowledge, vaccination history, vaccine perceptions and intentions, and responsibility toward others (refer to S1 Appendix for the interview protocol). The IRB-approved interview protocol provided exact question wording to be used with each participant but allowed for flexibility in the question sequence given the natural flow of discussion. A semi-structured interview format not only ensured that data were exhaustively collected from all participants, but also functioned to minimize interviewer effects. Follow-up questions and probes were also asked during interviews to gather a more elaborated understanding of participants’ thoughts. Interviews were approximately 45 minutes long and were audio-recorded with participants’ consent.

### Data management and approach to analysis

In order to facilitate data analysis across this large set of interviews we followed the data management and analytic approach used by Halcomb and Davidson [31]. In this approach, following the completion of each interview, the researchers reflected on their field notes expanding on their initial impressions and documented the major concepts and issues raised by the participants into summarizing field notes using Microsoft Word in order to avoid difficulties inherent to relying solely on audio recording transcriptions [32, 33]. Interviewers were trained to reflect upon the interviews and provided insights on participants’ health misperceptions, personal background, health information seeking, vaccine information access, influenza perceptions, vaccination intentions, vaccine perceptions, social norms, nonverbal behaviors, and any other issues or outcomes from the interview. During this post-interview reflection, the researchers turned back to the audio recording as needed to amend and revise their observations. They also identified and transcribed any direct illustrative quotes from the participants and included them in their field notes while using the raw audio files to corroborate and identify any discrepancies. This approach has been noted to help minimize interviewer bias and improve cross-case comparisons following the documentation of findings [31]. Neither the field notes nor the findings were returned to the participants for comments and corrections.

Following Halcomb and Davidson’s [31] approach, the field notes from all 76 interviews were then compiled into a 295-page compendium used for the primary analysis which employed grounded theory methods to analyze the data [34, 35, 36]. Grounded theory is an inductive process oriented toward building theory that is derived from the data under study [37]. The descriptive and explanatory power of this constructivist approach is advantageous in offering an in-depth understanding of current vaccine perceptions and how those perceptions were formed [38].

Concepts were developed mainly with *in vivo* coding that reflects the “vernacularity” of responses [39]. The *in vivo* approach typifies and labels concepts with the verbiage used by participants. This coding process differs from other coding practices such that it uniquely captures participants’ choice of language and nuanced meanings [40] and is particularly relevant to the Singapore context of this study where hybridized dialects have culminated into a version of English known colloquially as “Singlish” that is mixing words from other languages. In our study, *in vivo* codes provided an understanding of how our participants perceived influenza and vaccination (e.g., “influenza comes from wearing wet clothes” and “influenza vaccine kills the good cells in body”). Subcodes were also identified for some primary-cycle codes to furnish more details (e.g., subcodes for “influenza vaccine can cause side effects” listed examples of vaccine side-effects identified by participants). We involved comparisons of codes first within interviews and subsequently between interviews in order to ensure that all relevant themes were exhaustively catalogued and clustered.

## Results

76 participants (mean age = 73.19 years) were interviewed between January and April 2018 across Singapore (Table 1). The demographic profile of our participant pool is skewed toward females but has an ethnic composition similar to the latest Singapore population trends [41].

**Table 1:** Breakdown of participant characteristics (N = 76).

Gender	
Male	39.5%
Female	60.5%
Ethnicity	
Chinese	73.7%
Malay	13.2%
Indian	11.8%
Eurasian	1.3%
Vaccination History	
Have taken the influenza vaccine before at some point	59.2%
Have not taken the influenza vaccine before	31.6%
Unsure	9.2%

Our approach of constant comparison resulted in 132 first-level codes and we focused on 95 codes that reflected our study scope, which were then refined into 7 main themes and classified into two topic areas of perceptions about 1) the influenza disease, and 2) the influenza vaccine (Table 2).

**Table 2:** Typology of influenza disease and influenza vaccine perceptions.

**Topic**	Theme (number of codes)	Definition
**Perceptions about the influenza disease**	Familiarity with influenza (5)	This theme covers how well participants know about the influenza disease and its symptoms. Participants also describe their information source.
	Misperceptions about influenza (30)	This theme includes participants’ misperceptions about the influenza in terms of the disease’s characteristics, its transmission, its severity, and its prevention.
	Personal susceptibility to influenza (2)	Within this theme, participants describe how susceptible they perceive to be toward the influenza disease
**Perceptions about the influenza vaccine**	Familiarity with the influenza vaccine (19)	This theme covers how well participants know about the influenza vaccine and its symptoms. Participants also describe their information source.
	Misperceptions about the influenza vaccine (14)	This theme includes participants’ misperceptions about the influenza in terms of the vaccine’s characteristics, its risks, and its costs.
	Misperceptions about influenza vaccine usage (13)	This theme includes participants’ misperceptions about the influenza vaccine in terms of its usage. Here, participants share their perceptions on recommended and non-recommended users and point in times when to get vaccinated.
	Opinions about and barriers to influenza vaccine uptake (12)	Under this theme participants shared their opinions on the influenza vaccine and what barriers hinder them from taking the influenza vaccine up.

Participants were generally found to hold significant misperceptions about the influenza disease as well as the influenza vaccine. Most participants regarded influenza as a non-serious illness. Most participants also reported that they have received little or no information about influenza from healthcare providers, nor did they report having been informed about the risks and benefits of vaccination. Of the 76 participants, 27 participants (35.5%) indicated that they receive the influenza vaccine annually. Modeling on the interview structure, we analyzed disease- and vaccine-related factors from the perspective of Singaporean older adults and reported our major themes and findings below.

### Perceptions about the influenza disease

#### Familiarity with influenza

Influenza was largely unfamiliar among many participants with most acknowledging that they did not know much or anything about influenza. By and large the sample was unable to identify characteristics or determinant knowledge about the disease itself. One contributing factor is the use of multiple terms used to describe the disease and response. Many participants voiced confusion between the medical term “*influenza*” and the abbreviated term “*flu*” with many participants not recognizing it as the same disease. This was also observed with similar Mandarin Chinese translations (“influenza” = 流行性感冒 [or 流感 for short] versus “common cold” = 感冒). Terminology difference between “*influenza vaccine*” and the more colloquially used “*flu shot*” or “*flu jab*” also seem to obfuscate older adults’ ability to identify the disease and response: “*Influenza means some sickness…influences the lungs*. *Probably some kind of flu… I think influenza and flu are about the same family*, *quite the same*. *Flu may be passing flu*, *sometimes flu may come and go but influenza may be more serious*, *I don’t know*. *I think so*, *so I think*.” (Male, interview 5). Knowledge about influenza among participants was therefore rudimentary and not comprehensive.

Another point of confusion among participants was in their identification of influenza as being different from the common cold. This was illustrated by one participant who described influenza as manifesting in two forms—one that is less serious that involves mild symptoms such as cough and runny nose, and the other that is more serious and leads to fever or requires medical attention. Although less frequently reported among this sample, influenza was also confused with dengue fever.

Among participants who did report that they were familiar with influenza, only a few noted that they learned about the disease from mainstream news sources, especially during past epidemics: “*I read somewhere…A few years ago*, *Singapore had this epidemic*” (Female, interview 2) and “*I heard from the news… when they announce*, *what things happened…*” (Female, interview 78). The SARS epidemic in 2003 was cited as an event that has influenced Singaporeans in their attitude towards the flu and its vaccine: “*Last time Singapore got SARS… now they are quite alert*. *Last time Singapore got SARS*, *nobody knows*.” (Female, interview 14).

#### Misperceptions about influenza

While familiarity was low in general, the sample did provide many misperceptions about the disease which we divided into influenza characteristics, transmission, severity, and prevention. Each of these aspects is elaborated in the following sub-sections and illustrates our participants’ level of knowledge about influenza.

*Misperceptions about influenza characteristics*. Influenza was not universally understood by participants and many reports were not wholly inaccurate. Beyond the misunderstanding of disease terms, participants described influenza as (a) a global epidemic, (b) a type of sickness of the lungs, (c) an effect of blood circulation, and (d) seasonal during year-end, during festive periods such as Chinese New Year, “*rainy season*” (Male, interview 61), or at the end of the year: *“Usually end of the year I get flu; I don’t know why*. *Whole year round nothing happens but end of the year…”* (Female, interview 26).

*Misperceptions about influenza transmission*. Most participants reported incorrect knowledge about transmission modes of the influenza virus. Misperceptions about the source of influenza spread included (a) foreign countries, (b) animals, (c) the air, (d) the weather, (e) food types that are cooling, spicy, unclean, rich, or high in cholesterol, (f) wearing wet clothes, (g) a developing cold, (h) overexertion, and (i) toxins in the body.

*Misperceptions about influenza severity*. Understandings about the severity of influenza varied widely. 29 participants (38.2% of the sample) perceived influenza to be serious and fatal providing insights including, “*If [influenza is] not taken care of*, *you [might] get pneumonia*. *And you hear people dying of pneumonia*, *which is ridiculous*. *I mean*, *our former P[rime] M[inister] Mr*. *Lee Kuan Yew died of pneumonia too*” (Male, interview 41). On the other end of the spectrum, 26 participants (34.2% of the sample) regarded influenza as a minor illness of little concern: “*The flu is not so serious*. *Heart attack*, *very serious… diabetes*, *very serious… cancer*, *very serious*, *more important*” (Female, interview 28).

Influenza severity was also underestimated and attributed to internal factors such as (a) one’s immunity (“*[The severity of flu] depends on…immune system…but it seldom kills*” (Female, interview 26)), and (b) whether one self-medicates or practices self-care *(“[Influenza] will become serious only if I don’t see a doctor and self-medicate*, *particularly because I have allergies*” (Female, interview 28)). One participant saw influenza as an epidemic-level occurrence rather than as a frequently occurring viral activity: “*The degree of severity depends on the mortality rates*, *and how widespread it is*” (Male, interview 23).

*Misperceptions about influenza prevention*. While some participants identified vaccination and avoiding symptomatic persons, many reported that they engage in behaviors that are not likely to prevent influenza transmission. In general, participants frequently reported a strong immune system as the best prevention for influenza: *“If your immune system is good*, *you should not get it (influenza)”* (Male, interview 5). Many participants noted that influenza could be prevented through general positive health activities like maintaining a healthy diet, having sufficient sleep, exercising, and having good hygiene practices.

#### Personal susceptibility to influenza

Many participants considered themselves to be impervious to influenza infection. Personal susceptibility was mainly thought to depend on immunity level where only a weak immune could get ill from the virus. As one participant noted,

*If you’re tired*, *if you’re weak… we can catch the virus very fast*. *If your immune system is not very strong because sometimes you’re very tired and not [having] enough rest*, *that’s when the thing (flu virus) will attack*. *But if you’re healthy*, *sleep well*, *and you eat well*, *and you think you can fight against it… [but] sometimes you cannot*. (Female, interview 37)

Additionally, participants perceived personal immunity as something they have control over and is their own responsibility to maintain, for example through a) exercising, b) eating healthy, c) taking vitamins, d) avoiding (sick) people, or e) being happy.

### Perceptions about the influenza vaccine

#### Familiarity with the influenza vaccine

Participants who had heard about the influenza vaccine (n = 35), cited sources of information included (a) traditional news sources including television and newspaper reports (14.3%), (b) personal experiences (5.7%), (c) healthcare professionals (45.7%), and (d) doctor recommendations (51.4%). Some participants mentioned that they had heard about the influenza vaccine from healthcare professionals but did not receive personal recommendations to take the vaccine. Others said that they received little or no information about the influenza vaccine, and a minority reported having received recommendation to avoid the influenza vaccine from general practitioners and specialists (“*My doctor for arthritis say it’s not necessary*. *He says it’s better to get more rest than to go for these vaccines*”–Female, interview 34). Participants reported that dialogue with family and friends led to an exchange of information about the influenza vaccine, barriers to vaccination, and personal recommendations to take the vaccine: “*My whole family… because we have family gatherings often and the last ones I told all my siblings [to get the influenza vaccine]*” (Female, interview 11) and “*They do say they take this kind of (influenza) vaccination before traveling or something*, *so that brings to me in mind that you need it when*, *you know*, *go traveling*” (Female, interview 69).

#### Misperceptions about the influenza vaccine

Participants’ vaccine perceptions were found to be broadly focused on influenza vaccine characteristics, risks, and cost.

*Misperceptions about influenza vaccine characteristics*. The function of the influenza vaccine was largely unfamiliar among participants. The influenza vaccine was commonly misperceived as a travel vaccine only intended for people leaving the country for places with lower hygiene standards: *“For me I only go [overseas] for holidays*. *Most of the time*, *like third-world countries then I go for jab*. *Other than that*, *I don’t go for jab”* (Male, interview 1). Hygienic standards, sexually transmitted diseases, and even homosexuality were referenced as reasons to be vaccinated against influenza, with one participant noting that “*Because other countries have many gays [travelers should get the vaccine]*” (Male, interview 65) to protect themselves from potential infection. This stigmatizing sentiment was also coupled with perceptions that such cases do not occur in Singapore as “*Singapore not so much*” (Male, interview 65). Another participant reported that the influenza vaccine “*protects against everything*” (Male, interview 23). This implies confusion about the function of the influenza vaccine and perhaps vaccines in general—that vaccines are a universal preventive measure rather than targeted at specific diseases.

Misperceptions and concerns over vaccine safety also arose among some participants. Two participants reported that the influenza vaccine contains germs or viruses from animals that would likely have deleterious effects on humans: *“Vaccination comes from animals and some of the thing [diseases] will come into contact with humans*. *Vaccine is something [that] comes from animals*, *not safe”* (Male, interview 61). Not all misperceptions held negative connotations however as a participant contrastingly believed that the vaccine contains vitamins and recommended it for this benefit: “*I recommend my friend [to] take this because [of the] good vitamins*” (Female, interview 33).

*Misperceptions about influenza vaccine risks*. Apart from safety concerns over vaccine content, participants reported misperceptions that the influenza vaccine (a) weakens the body’s immune system, and (b) causes the disease: *“People don’t have the sickness and then get vaccinated and then they get the sickness*. *Some of them who got vaccinated got the illness through the vaccination”* (Male, interview 5). These perceptions were commonly drawn from second-hand experiences where participants noted hearing about side effects from other vaccine users. One participant was also apprehensive about taking the seasonal influenza vaccine as it is newly formulated each year. As one participant noted, “*I’m not comfortable with that kind*, *keep on testing and testing*, *they also didn’t have enough long-time research… too new*” (Male, interview 74).

*Misperceptions about influenza vaccine cost*. Perceived financial cost played a significant role in participants’ intentions to get the influenza vaccine. Few participants were aware of government support programs for getting vaccinated and most were concerned about the financial burden that comes with the decision to vaccinate. The misperception that the influenza vaccine is expensive was widely prevalent among participants: “*[Vaccination is] necessary but it costs me money*. *One injection [is] S$40… expensive*” (Male, interview 70).

#### Misperceptions about influenza vaccine usage

Beyond misperceptions about the influenza vaccine, the study explored participants’ opinion of who should take the influenza vaccine and when it should be taken.

*Perceptions about non-recommended users of influenza vaccine*. A healthy and active lifestyle was thought to be a preventive measure against influenza to the extent that young, active individuals with strong immunity were perceived to not require vaccination: *“Strong people are strong so they don’t need [the influenza vaccine]”* (Female, interview 20). Accordingly, a participant concluded: “*Only the old people need [the vaccine]*. *My daughter doesn’t need*” (Female, interview 4).

*Perceptions about recommended users of influenza vaccine*. Many groups were reported to be in-need of vaccination against influenza including (a) those who travel, (b) those who work in the food industry, (c) those who work in crowded areas, and (d) those with skin diseases. India, China, the United States, and less-developed nations were listed as countries that require influenza vaccination for travelers.

*Misperceptions about when to get influenza vaccine*. Many participants reported that the vaccine was not intended for regular or periodic use. Some believe that the vaccine is only of value for use before or during epidemic-level events: *“The SARS or H1N1*, *once they come*, *cannot stop because Singapore is a tourist [hub]*. *It’s a good prevention”* (Male, interview 38). Another woman echoed this sentiment saying, “*I don’t want any [vaccines]… unless there is an urgent need*. *Maybe it’s a major influenza crisis*” (Female, interview 34). Others noted that the vaccine is only intended for use as a treatment once an individual has influenza symptoms: “*The vaccine prevents the influenza from getting worse*, *and for the flu to relapse*” (Male, interview 12). Participants also confused the purpose of vaccines with antiviral drugs that are used for treating influenza infections rather than as a preventive measure: “*Vaccines kill the virus*” (Male, interview 13). Antiviral drugs serve to lessen disease severity and reduce the probability of complications when taken in the first two days of infection; therefore, antiviral drugs do not have the same function and cannot be substitutes for vaccines, according to the Centers for Disease Control and Prevention [42].

#### Opinions about and barriers to influenza vaccine uptake

Despite proven vaccine effectiveness against influenza infection, health officials face tangible and intangible challenges in encouraging vaccine uptake. While the influenza vaccine is one of the most affordable vaccines ranging between 30 and 40 Singapore Dollars (approximately 22 to 29 US Dollars), most participants wanted the vaccine to be cheaper or even cost-free. Participants reported that the government should provide more financial support for older citizens: “*I think people will say it’s expensive for Singaporeans who don’t have enough money*. *The government has to subsidize a bit for the poor people*” (Male, interview 61). One participant also voiced concerns that doctors held profit interests as reasons for why they would recommend the vaccine in the first place. Vaccination was also regarded as an inconvenience given the need to travel and the long waiting hours at polyclinics. Other barriers mentioned included (a) a fear of injections, side effects and pain, (b) a lack of knowledge about the influenza vaccine, (c) religious objections, and (d) a dislike for taking any form of medication.

## Discussion

Influenza poses a significant public health challenge in Singapore with high morbidity and mortality particularly among older adults. However, vaccine uptake is observed to be low despite strong medical recommendations for older adults to regularly vaccinate against the seasonal influenza epidemics. This study thus explored older adults’ influenza disease and vaccine misperceptions with the aim of understanding barriers to vaccination and how these barriers may be remedied to improve this population’s health and well-being. Our results demonstrated poor health knowledge among our participants that is indicative of the contributing effects of misperceptions on Singaporean older adults’ hesitancy toward influenza vaccination. We discuss possible reasons for these misperceptions and how communication can enhance older adults’ health knowledge and ultimately their uptake of the influenza vaccine.

### Perceptions about the influenza disease

Participants’ misunderstanding of disease terms (“influenza” versus “flu”) and the characteristics of influenza demonstrate partial knowledge. Two participants noted the similarities of influenza and cold symptoms but claimed that they have not experienced or been infected with influenza. However, previous studies noted that non-health professionals cannot clearly differentiate influenza from the common cold [43]. Furthermore, in its precise definition, influenza is not a “sickness of the lungs” but rather a respiratory disease that leads to fever and can infect the lungs which may also lead to the development of pneumonia for at-risk individuals.

Participants generally have some rudimentary knowledge of influenza, but also are likely to conflate symptoms with other diseases. The inability to correctly recognize influenza may encourage incorrect prevention and treatment practices among older adults. Further misperceptions about how influenza spreads and how it can be prevented pose significant concerns for this at-risk population as they neither understand the severity of the disease nor have the health knowledge to facilitate adoption of adequate practices to stop the spread of influenza. Therefore, misperceptions about influenza may inadvertently be putting this group at further risk to ancillary diseases and accidents and warrant future engagement regarding influenza transmission and prevention.

Perceptions of good health status and personal immunity as reasons for an individual’s influenza infection were prevalent among participants. These misperceptions are consonant with Sundaram et al.’s [27] findings that healthcare workers perceived influenza “as a routine illness causing temporary inconvenience but no lasting harm” (p. 1999) and with those of some healthcare workers (as found by [44, 45]). This consensus indicates that more communication about the non-relationship between immunity and influenza infection is needed to redress the misperceptions among older adults in Singapore. The prevalence of this “*strong immunity and therefore no infection*” misconception among both older adults and healthcare workers is concerning, particularly when provider–patient communication may propagate bad health knowledge and impede health promoting behaviors. Influenza communication could therefore emphasize on older adults’ susceptibility and likelihood of developing health complications from influenza to counter this barrier to vaccination.

Further aspects should be taken into account for future communication strategies that aim to reach a target group of 65+ years of age. Older adults face significant challenges concerning memory recall of knowledge-based appeals, and this is exacerbated by age as linear declines in fluency, memory, and perceptual speed are observed as people grow older [46]. Older adults also tend to heuristically process complex information such as those related to health as their cognitive abilities decline with age [46]. Finucane [47] pointed out that older adults are inclined to rely more on emotion-evoking information and suggested that risk information may be portrayed with numeric displays, visuals and in narratives to enhance likelihood of message processing and increase message salience among older audiences. Orel, Spence, and Steele [48] proposed providing factual information to counteract misperceptions and discussing old age vulnerabilities to increase perceived disease susceptibility. Cummings [46] added that written instructions should be accompanied with verbal consultations with healthcare professionals to ensure message comprehension. Message tailoring to older audiences has also been recognized as an effective strategy in increasing information salience and can contribute to reducing information exposure barriers [49]. These recommendations can be incorporated into future communication to improve knowledge about influenza. Sparks and Nussbaum [50] suggested tailoring interpersonal messages to older adults’ personal characteristics and information processing abilities to improve health knowledge. While their recommendation was oriented toward older cancer patients, this advice is not any less useful for encouraging vaccination among older adults now that this group’s primary misperceptions about the influenza disease have been systematically identified in this study.

### Perceptions about the influenza vaccine

Our participants also held various misperceptions about the influenza vaccine. Of which, the most common misperception is that the influenza vaccine functions as a travel vaccine. Singapore has an extensive history of disease entering from international travelers, and this seems to influence perceptions that within Singapore, there is little viral spread among residents. News headlines commonly reiterate to get vaccinated when “travelling for your year-end holiday” or before beginning “studies overseas” [13]. While laudable advice, such stories may implicitly contribute to a perpetuated misperception that vaccines including those against influenza should be taken only before traveling and not as routine practice. This suggests that how communication is framed regarding vaccines plays an influential role in shaping perceptions. Therefore, beyond the recommendation that the influenza vaccine should be taken before traveling, vaccination as a routine practice needs to be emphasized to achieve herd immunity and prevent future epidemics.

We noted participants’ confusion of the influenza vaccine with antiviral drugs that are taken to treat influenza infections. This further signifies poor vaccine knowledge among older adults that may explain the low and delayed vaccine uptake (i.e., taking the vaccine only after signs of infection). Poor vaccine knowledge has been recognized as one of the barriers to influenza vaccination [51]. Our study adds that confusion between the functions of vaccines and antivirals is a form of poor vaccine knowledge. Vaccine communication should thus emphasize on the functionality of vaccines, that is, vaccines should be taken before an infection as a preventive measure.

Participants raised further concerns over the safety of the influenza vaccine with regards to its content and periodic manufacturing. This corroborates the SAGE Working Group’s Vaccine Hesitancy Determinants Matrix, in which the introduction of new formulation or new recommendations for existing vaccines was identified to influence hesitancy [52]. Participants’ concerns may be alleviated with communication about vaccine safety (i.e., does not contain germs or zoonotic viruses) from multiple points such as the local government, health organizations, and health practitioners. More comprehensible explanations are necessary to instill knowledge about the mutability of influenza viruses and the need for vaccine manufacturers to produce new vaccines every year to protect users from circulating mutated influenza strains.

Misperceptions about the influenza vaccine are suggested to be exacerbated by inadequate communication about the vaccine. Participants who reported having heard about the influenza vaccine cited doctors as their primary source for vaccine information. Most other participants reported that they have not received any information from their doctors nor had they been recommended the vaccine by healthcare professionals. Even if this is not the actual case—even if these individuals have been informed by healthcare professionals about the influenza vaccine, the message simply is not getting through satisfactorily. This highlights a significant communication gap between doctors and older patients and warrants reinvigorated efforts to adequately engage and ensure that older adults have access and attend to influenza vaccine communication from their healthcare providers. Older patients tend to exhibit less assertive and more paternalistic attitudes toward providers [53] which suggests that older adults may be more willing to comply with direct vaccination recommendations from healthcare professionals than their younger counterparts.

Health engagement initiatives should therefore begin with reinforcement through interpersonal channels via direct communication from healthcare professionals and accompanied by reiterative written instructions and materials that outline the known risk-benefit relationships between influenza vaccination and disease prevention. Many misperceptions may be corrected through adequate direct recommendations from healthcare professionals. Stronger communication efforts to dispel identified vaccine misperceptions and promote vaccine knowledge are warranted.

### Limitations and future directions

This study has three limitations that must be acknowledged. First, even though the use of novice interviewers and executive summaries may have been an outcome of limited study resources, our data analysis relied on interviewers’ extensive training, executive summaries and audio playbacks rather than full interview transcripts. This approach introduced the issue of subjective data selection and filtering through interviewers and hence the interpretation of incomplete or reduced qualitative data. Second, the use of nonprobability sampling techniques in recruiting participants limits the generalizability of our qualitative findings. Future replications using stratified sampling techniques are warranted to validate the findings from this present study. Last, language differences are inherent in this study that is conducted in polylingual Singapore where most older adults speak more often in their native language than in English. Despite our interviewers’ language proficiency, interpreting and translating any Mandarin Chinese into English nonetheless pose challenges to the validity of our study findings.

Our study is the first qualitative research in Singapore to identify a typology of its older adults’ misperceptions about the influenza disease and its vaccine. Our findings provide public health practitioners an insight on the complexity of vaccine perceptions among one of the most vulnerable populations. Through this study, we hope that health professionals may be better informed of older adults’ concerns about influenza vaccination and in turn be able to refine their personal communication with this population in encouraging vaccination.

## Conclusion

Improving health knowledge and ultimately, public uptake of preventive measures like vaccination remain among the most significant public health challenges in the 21^st^ century. In developed countries like Singapore, issues of access to preventive healthcare like the influenza vaccine are minor—older adults can easily obtain and afford the influenza vaccine. What remains is a deficiency of public awareness, adequate knowledge, motivation, and execution in proactive health behaviors including taking the seasonal influenza vaccine. Reinvigorated efforts of targeted health communication can bridge this gap and move us forward in protecting our older communities from disease.

## Supporting information

S1 Appendix Interview protocol. (DOCX)
