# Supplementary material for: A typology of beliefs and misperceptions about the influenza disease and vaccine among older adults in Singapore
Source: PLoS One. 2020 May 6;15(5):e0232472. doi: 10.1371/journal.pone.0232472 (PMC7202625; doi:10.1371/journal.pone.0232472)
Supplement: S1 Appendix — (DOCX) [file pone.0232472.s001.docx]

**Appendix: Interview protocol**

1. Can you introduce yourself?
2. What is the first word that comes to mind when you hear the word influenza?
3. What is the first word that comes to mind when you hear the word vaccine?
4. How do you usually seek health information?
5. Have you had any problems when seeking health information?
6. Have you come across any information that have shaped your views on vaccination?

**[Facilitator to provide baseline knowledge on influenza disease for Q7 and Q8]**

1. Have you experienced these influenza symptoms?
2. Has any medical professional ever recommended you to get vaccinated for influenza?

**[Facilitator to provide baseline knowledge on influenza vaccine for Q9]**

1. Have you taken the influenza vaccine previously?
2. Do you intend to get vaccinated against influenza?
3. How do you typically seek healthcare treatment? (e.g., from Western doctors, TCM consultants, etc.)
4. Has your primary healthcare provider given you any information about the influenza vaccination?
5. Who should get vaccinated for influenza? Why?
6. What kind of information have you gotten about the influenza vaccination? Where and what have you heard or read about it?
7. Do you have any concerns regarding vaccinations?
8. In Singapore, influenza vaccines typically cost between $30-$40. How do you feel about this pricing for vaccines?
9. How do you think MOH is doing with respect to influenza vaccination?
10. Are vaccines effective in preventing influenza?
11. Are you able to get vaccinated?
12. Do you think influenza is a serious sickness?
13. How likely do you think you will be infected with influenza?
14. Has any of your family members been vaccinated against influenza previously?
15. Do you feel a responsibility toward the health of your loved ones and your community?
16. What do your friends think about getting vaccinated against influenza?
17. How many elderly Singaporeans do you think have vaccinated against influenza?
18. Do you remember any past events that have influenced your decisions about getting vaccinated?
